# Latent Profile Analysis and Influencing Factors of Clinical Nurses’ Evidence‐Based Practice Implementation: A Cross‐Sectional Study

**DOI:** 10.1155/jonm/8783451

**Published:** 2026-06-25

**Authors:** Yilin Chen, Jie Wang, Yi Zhang, Xiuzhu Cao, Linfang Zhao

**Affiliations:** ^1^ Nursing Department, Sir Run Run Shaw Hospital, School of Medicine, Zhejiang University, Hangzhou, Zhejiang, China, zju.edu.cn

**Keywords:** evidence-based practice, evidence-based practice belief, evidence-based practice organizational culture and readiness, nurse

## Abstract

**Aim:**

The purpose of this study was to investigate the current status and potential profiles of clinical nurses’ implementation of evidence‐based practices (EBPs), explore influencing factors across different profiles, and provide a basis for developing strategies to enhance clinical nurses’ EBP competence.

**Design:**

A cross‐sectional study.

**Methods:**

From January to March 2025, a convenience sampling method was used to select 289 clinical nurses from 24 provinces across China. A survey was conducted using a demographic questionnaire and the three EBP scales–short version to assess implementation. Latent profile analysis identified distinct latent profiles of EBP implementation among Chinese clinical nurses, and multivariate logistic regression examined the factors influencing profile membership.

**Results:**

The results revealed three latent profiles of EBP implementation among clinical nurses: low participation type (15%), moderate participation type (27%), and high participation type (58%). Multivariate logistic regression analysis indicated that EBP beliefs, EBP organizational culture, and readiness were the primary influencing factors of EBP implementation.

**Conclusion:**

This study demonstrates that Chinese clinical nurses generally exhibit positive engagement in EBP implementation. EBP beliefs, EBP organizational culture, and readiness are key factors influencing implementation. Efforts should focus on fostering EBP beliefs while simultaneously providing a supportive implementation environment to ensure sustainable EBP.

**Summary**
• Reporting method
◦ The results of this study were reported in accordance with the Strengthening the Reporting of Observational Studies in Epidemiology (STROBE) guidelines.
• Impact
◦ The findings of this study will contribute to identifying the categories and heterogeneity of clinical nurses’ EBP practices, providing a basis for nursing educators and managers to develop corresponding strategies to enhance nurses’ EBP.
• Patient or public contribution
◦ All participants were nurses who completed an electronic questionnaire.

## 1. Introduction

Despite the recent substantial increase in evidence synthesis, translating effective findings into clinical practice persists as a long‐standing challenge in the healthcare domain, commonly denoted as the “knowledge‐to‐practice” gap. This disparity between theoretical knowledge and real‐world application represents a crucial concern in evidence‐based practice (EBP). In the field of healthcare, only 14% of research findings are translated into clinical practice, with an average implementation period of 17 years [1]. EBP is a problem‐solving approach to healthcare delivery that integrates the best evidence from a body of research with a clinician’s expertise and a patient’s preferences and values to make the best decisions about patient care [2]. EBP plays a crucial role in bridging the gap between theory and practice within the healthcare domain, reducing health inequalities and improving the quality of healthcare services. In 2017, the World Health Organization underscored that promoting EBP should be a priority strategy to promote the improvement of healthcare [3].

## 2. Background

As facilitators of healthcare, nurses are central to EBP. They can provide optimal care decisions for patients, enhance the quality of nursing care, and drive the development of the nursing discipline. The rise of EBPs in nursing marks a transformation of the nursing discipline from an experience‐oriented approach to one based on scientific decision‐making. Traditional nursing models often rely on individual experience or established conventions, whereas evidence‐based nursing provides scientific support for nursing practice through systematic retrieval, evaluation, and application of the best evidence. This approach has achieved favorable outcomes in terms of improving clinical results. The application of EBP in adult patients in the intensive care unit reduced the incidence of artificial airway–related pressure injuries by 11.88% [4]. In maintenance hemodialysis patients, the application of EBP for puncture management can reduce the incidence of arteriovenous fistula hematoma and reduce treatment interruption [5]. Hu et al.’s study on patients with upper gastrointestinal bleeding in the emergency department revealed that the complication rate in the EBP group was 5%, which was significantly lower than the 16.7% in the control group, with quality of life also significantly higher in the EBP group [6]. In addition, research conducted on liver transplant patients demonstrated that those who received preoperative EBP had a significantly reduced postoperative infection rate [7]. In addition to the benefits it provides to the healthcare system, the WHO emphasizes that EBP itself can increase nurses’ job satisfaction and empowerment, which can improve skills to integrate patient preferences into practice, as well as support for professional growth and continuous career development through expert roles [3]. The implementation of EBP not only improves patient outcomes and enhances healthcare quality but also promotes positive professional empowerment for nurses.

Globally, nurses’ practices are a persistent challenge, characterized by insufficient knowledge and skills and low behavior conversion rates in terms of EBP, which are particularly prominent in low‐ and middle‐income countries [8–10]. A scoping review focusing on low‐ and middle‐income countries revealed that nurses have a low level of familiarity and awareness with EBP, with more than 60% of the studies characterizing nurses’ EBP knowledge and skills as moderate, low, or insufficient, and approximately 84% of the studies described the implementation of EBP in healthcare settings as moderate, low, poor, or suboptimal [11]. Research conducted in China has yielded similar findings. Studies conducted by Yini et al. and Yun et al. have indicated that although nurses generally hold positive attitudes toward evidence‐based nursing, their scores for knowledge and skills are significantly low [12, 13]. This fragmented state of “high willingness but low ability” severely restricts the implementation of EBP in nursing practice.

In China, research on nurses’ implementation of EBP has several limitations, such as small sample sizes and uneven geographical distribution, making it difficult to comprehensively reflect the overall status of EBP among Chinese nurses. In particular, there is a lack of nationwide survey data, which prevents us from accurately assessing the true level of EBP among Chinese clinical nurses and its regional variations. To address this gap, this study carried out a nationwide survey and employed latent profile analysis to identify different ability subgroups among nurses and conducted an in‐depth analysis of the characteristic differences among these subgroups. This research provides an important theoretical basis and practical guidance for the subsequent development of targeted stratified training programs and the establishment of a scientific organizational support system, thereby comprehensively enhancing the EBP capabilities of Chinese nurses.

## 3. The Study

This study aims to develop latent profiles of EBP implementation among clinical nurses and explore the factors associated with different profiles. The research questions were as follows: (1) Is there heterogeneity in the implementation of EBP among clinical nurses? and (2) Which factors are associated with the distinct profiles of EBP implementation among clinical nurses?

## 4. Methods

### 4.1. Study Design

A quantitative cross‐sectional design was used for this study.

### 4.2. Participants

A convenience sampling method was employed to conduct a questionnaire survey among 289 nurses from 48 regions across 24 provinces in China between January and March 2025. The inclusion criteria were as follows: (1) nurses who held a valid nursing practice qualification certificate and (2) nurses who provided informed consent and voluntarily participated in the survey. The exclusion criteria were as follows: (1) nurses who were absent from clinical practice because of illness, maternity, or personal leave and (2) nurses who were currently studying as students or taking refresher courses.

### 4.3. Instruments

#### 4.3.1. Demographic Questionnaire

The data included gender, age, region, hospital level, type of hospital, educational level, professional title, work position, and work years.

#### 4.3.2. Three EBP Scales–Short Version

The three EBP scales–short version was developed by Professor Melnyk et al. [14] and localized into Chinese by Weihua et al. [15]. It consists of three subscales: the EBP Beliefs Scale, the EBP Implementation Scale, and the EBP Organizational Culture and Readiness Scale. Each subscale contains 3 items and uses a 5‐point Likert scale, with 1 representing “strongly disagree” and 5 representing “strongly agree.” The Cronbach’s *α* coefficients for the three subscales are 0.81, 0.89, and 0.87, respectively. This scale demonstrates good operability because of its simple and easily understandable items, which facilitate comprehension and acceptance.

### 4.4. Data Collection

Data were collected using the “Wenjuanxing” online platform. Researchers established contact with members of the Cross‐Strait Medicine and Exchange Association across various regions, who distributed the questionnaire link to clinical nurses. Prior to initiating the questionnaire, the participants were clearly informed about the purpose and significance of the survey. All participants provided informed consent and completed the questionnaire anonymously. To ensure a high response rate and data completeness, the questionnaire was designed with mandatory questions, skip logic, and a restriction that prevented submission in the case of missing responses. To avoid duplicate submissions and ensure data quality, the “Wenjuanxing” backend was configured to allow only one submission per user account, with no modifications permitted after submission. Questionnaires completed in less than 1 min with obvious response patterns were excluded as invalid.

### 4.5. Statistical Analysis

Latent profile analysis was performed on the three subscales of the EBP scale using Mplus 8.3 software. The optimal model was selected on the basis of evaluations of model fit indices, including the Akaike information criterion (AIC), Bayesian information criterion (BIC), sample‐adjusted BIC (aBIC), entropy value, bootstrapped likelihood ratio test (BLRT), and Lo–Mendell–Rubin likelihood ratio test (LMRT). Specifically, smaller AIC, BIC, and aBIC values indicate better model fit, and an entropy value closer to 1 indicates more accurate classification. A *p* value < 0.05 suggests that the k‐class model fits better than the (k−1)‐class model. Statistical analyses were conducted using SPSS 25.0 software with two‐tailed tests and a significance level (*α*) set at 0.05. Measurement data conforming to a normal distribution were described using means and standard deviations (SDs), and categorical data were described using frequencies and percentages. Multivariate regression analysis was used to explore the factors influencing EBP implementation.

### 4.6. Ethical Considerations

The study was conducted in accordance with the Declaration of Helsinki and followed good scientific practices and principles of research integrity. Ethical approval was obtained from the Ethics Committee of Sir Run Run Shaw Hospital (No. 20240240). Before the study began, all the participants were informed about the purpose and process of the study, and their participation was voluntary.

## 5. Results

A total of 289 questionnaires were distributed, and all 289 were recovered as valid, resulting in an effective response rate of 100%.

### 5.1. Sociodemographic Characteristics of Nurses

Among the 289 participants, 269 (93.1%) were women and 20 (6.9%) were men. The average age was 34.62 ± 7.08 years, and 83.4% had a bachelor’s degree (Table 1).

**TABLE 1:** Sociodemographic characteristics of clinical nurses.

Items	Total sample (*n* = 289) M ± SD/*n* (%)	Class 1 (*n* = 43) M ± SD/*n* (%)	Class 2 (*n* = 78) M ± SD/*n* (%)	Class 3 (*n* = 168) M ± SD/*n* (%)	*χ* ^2^/*Z*	*p*
Age	34.62 ± 7.08	32.77 ± 5.76	33.72 ± 6.86	35.51 ± 7.37	6.255	0.044
Work years	12.24 ± 7.90	10.07 ± 6.48	11.36 ± 7.73	13.20 ± 8.18	6.066	0.048
Gender					6.873	0.032
Male	20 (6.9%)	7 (16.3%)	4 (5.1%)	9 (5.4%)		
Female	269 (93.1%)	36 (83.7%)	74 (94.9%)	159 (94.6%)		
Regions					2.340	0.310
North	103 (35.6%)	11 (25.6%)	28 (35.9%)	64 (38.1%)		
South	186 (64.4%)	32 (74.4%)	50 (64.1%)	104 (61.9%)		
Type of hospital					2.123	0.346
General hospitals	238 (82.4%)	38 (88.4%)	66 (84.6%)	134 (79.8%)		
Specialized hospitals	51 (17.6%)	5 (11.6%)	12 (15.4%)	34 (20.2%)		
Hospital level					0.802	0.670
Tertiary hospitals	260 (90.0%)	39 (90.7%)	72 (92.3%)	149 (88.7%)		
Level II hospitals	29 (10.0%)	4 (9.3%)	6 (7.7%)	19 (11.3%)		
Professional title					1.552	0.817
Elementary	122 (42.2%)	21 (48.8%)	34 (43.6%)	67 (39.9%)		
Intermediate	120 (41.5%)	16 (37.2%)	33 (42.3%)	71 (42.3%)		
Senior	47 (16.3%)	6 (14.0%)	11 (14.1%)	30 (17.8%)		
Work position					9.098	0.168
Registered nurse	208 (72.0%)	37 (86.0%)	55 (70.5%)	116 (69.1%)		
Nurse educator	15 (5.2%)	1 (2.3%)	3 (3.8%)	11 (6.5%)		
Specialist nurse	29 (10.0)	2 (4.7%)	12 (15.4%)	15 (8.9%)		
Head nurse	37 (12.8)	3 (7.0%)	8 (10.3%)	26 (15.5%)		
Educational level					3.179	0.528
College	14 (4.8%)	1 (2.3%)	2 (2.6%)	11 (6.5%)		
Bachelor	241 (83.4%)	38 (88.4%)	65 (83.3%)	138 (82.2%)		
Master and above	34 (11.8)	4 (9.3%)	11 (14.1%)	19 (11.3%)		

*Note:* Class 1, low participation type; Class 2, moderate participation type; Class 3, high participation type.

### 5.2. Latent Profile Analysis of Nurses’ EBP Implementation

**FIGURE 1:**
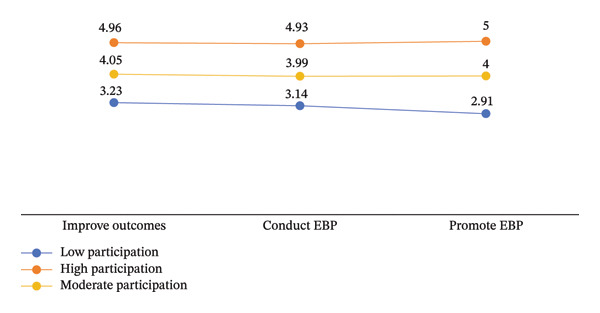
Three latent profiles of nurses’ EBP implementation.

On the basis of the model fitting results in Table 2, the optimal number of latent profiles was ultimately determined to be three. Although the entropy value of the one‐class model is equal to 1, its AIC, BIC, and aBIC values are higher. In contrast, the models with four and five classes exhibit lower AIC, BIC, and aBIC values, but their entropy values are also lower. Therefore, through comprehensive analysis, when the number of latent profiles is three, the AIC, BIC, and aBIC values are relatively small; the entropy value is greater than 0.9; and *p* values of both the LMRT and the BLRT are less than 0.05. These results indicate a high degree of model fit and accuracy. In addition, the class probabilities are reasonable when 3 classes are selected; thus, the 3‐class model was judged to be the most suitable for interpretation and in‐depth analysis. Based on the scores of each item across the 3 identified classes of nurses’ EBP implementation, they were named “Low Participation Type” (15%), “Moderate Participation Type” (27%), and “High Participation Type” (58%) (Table 2) (Figure 1).

**TABLE 2:** Model fitting results of the latent profile analysis model for nurses’ EBP implementation.

Classes	AIC	BIC	aBIC	Entropy	LMRT	BLRT	Latent class distribution rate (%)
1	2021.954	2043.952	2024.925	1.000	NA	NA	—
2	1410.050	1446.714	1415.003	0.960	< 0.001	< 0.01	0.39/0.61
3	909.545	960.875	916.479	0.999	< 0.001	< 0.01	0.15/0.58/0.27
4	813.908	879.904	822.823	0.996	< 0.001	< 0.01	0.05/0.58/0.27/0.10
5	785.390	866.051	796.285	0.997	< 0.001	< 0.01	0.04/0.57/0.26/0.10/0.03

### 5.3. Multivariate Logistic Regression Analysis of EBP Implementation

With Class 3 as the reference group, a multivariate logistic regression analysis was performed to determine the predictive factors associated with the EBP implementation of clinical nurses. The predictive factors for the EBP implementation of clinical nurses, including EBP beliefs, EBP organizational culture, and readiness, are shown in Table 3.

**TABLE 3:** Results of multivariate logistic regression analysis of EBP implementation.

Variables	Class 1					Class 2				
	*β*	SE	*p*	OR	95% CI	*β*	SE	*p*	OR	95% CI
EBP beliefs	−2.286	0.336	0.000	0.102	(0.053, 0.197)	−1.618	0.268	0.000	0.198	(0.117, 0.336)
EBP organizational culture and readiness	−1.588	0.234	0.000	0.204	(0.129, 0.323)	−0.780	0.163	0.000	0.458	(0.333, 0.631)
Age	0.105	0.225	0.640	1.111	(0.715, 1.726)	0.007	0.158	0.966	1.007	(0.738, 1.373)
Work years	−0.198	0.208	0.340	0.820	(0.546, 1.232)	−0.090	0.141	0.522	0.914	(0.693, 1.205)
Regions										
North	−0.578	0.795	0.467	0.561	(0.118, 2.662)	−0.247	0.552	0.655	0.781	(0.265, 2.306)
Gender										
Male	−0.277	1.436	0.847	0.758	(0.045, 12.665)	−1.104	1.180	0.350	0.332	(0.033, 3.350)
Type of hospital										
General	1.228	1.208	0.310	3.414	(0.320, 36.459)	0.200	0.789	0.800	1.222	(0.260, 5.739)
Hospital level										
Tertiary	−1.642	1.431	0.251	0.194	(0.012, 3.198)	−0.805	1.025	0.432	0.447	(0.060, 3.333)
Professional title										
Elementary	−3.747	2.081	0.072	0.024	(0.000, 1.392)	−1.479	1.358	0.276	0.228	(0.016, 3.261)
Intermediate	−3.713	1.622	0.022	0.024	(0.001, 0.586)	−1.890	1.012	0.062	0.151	(0.021, 1.099)
Work position										
Registered nurse	−0.109	1.669	0.948	0.897	(0.034, 23.613)	−0.324	0.892	0.716	0.723	(0.126, 4.152)
Nurse educator	−1.508	2.411	0.532	0.221	(0.002, 24.952)	−0.876	1.376	0.525	0.417	(0.028, 6.185)
Specialist nurse	−1.317	1.920	0.493	0.268	(0.006, 11.538)	0.217	1.030	0.833	1.243	(0.165, 9.356)
Educational level										
College	−2.549	2.777	0.359	0.078	(0.000, 18.066)	−2.471	1.655	0.135	0.085	(0.003, 2.165)
Bachelor	0.307	1.331	0.817	1.360	(0.100, 18.449)	−0.250	0.911	0.784	0.779	(0.131, 4.647)

*Note:* Class 1, low participation type; Class 2, moderate participation type.

## 6. Discussion

Previous studies have often analyzed nurses’ EBP implementation as a homogeneous group, overlooking individual heterogeneity. In this study, clinical nurses were categorized into three distinct groups on the basis of their EBP implementation characteristics. These three groups exhibited clear differences in gradient, reflecting the heterogeneous nature of nurses in terms of EBP implementation. The results revealed that the low participation group accounted for 15%, the moderate participation group accounted for 27%, and the high participation group accounted for 58%. Unlike the findings of previous studies, the findings of this study demonstrate a more positive engagement of nurses in EBP implementation, which stands in stark contrast to the results of a scoping review conducted in low‐ and middle‐income countries, which indicated that 84% of studies described EBP implementation in healthcare settings as moderate, low, poor, or suboptimal [11]. Furthermore, our results are also higher than those reported in another study from Ethiopia, where the pooled prevalence of EBP uptake among nurses was 53% [16]. The positive participation of nurses in EBP observed in our study, which differs from the predominantly negative participation reported in most previous studies, may be attributed to the active national promotion of high‐quality nursing services and the popularization of evidence‐based nursing practice in recent years. Medical institutions across various regions have successively established EBP centers, which may effectively increase nurses’ enthusiasm and capabilities in EBP through measures such as specialized training programs and the establishment of evidence‐based nursing groups. In addition, with the advancement of information technology, nurses now have more convenient access to the latest research evidence, which may also provide strong support for their active participation in EBP. However, most of the samples (90%) in this study were from tertiary hospitals, and the findings may not fully reflect the actual situation of nurses in primary‐level healthcare institutions. Tertiary hospitals typically have more comprehensive resource support, a stronger academic atmosphere, and a more systematic training system, all of which may create more favorable conditions for nurses to engage in EBP.

Further analysis of the demographic characteristics of the three groups revealed significant differences in age, work years, and gender between the different groups, which may be important reasons for the gradient differentiation in their EBP implementation. The results of this study revealed that older nurses may perform better in EBP practices, which may be related to their relatively rich clinical experience and greater ability to translate evidence‐based knowledge into practical operations. However, these findings contrast with those of existing studies that show that older nurses have weaker EBP implementation [9, 17]. Moreover, the results of this study revealed that nurses with more years of experience were better able to implement EBP, which is consistent with the results of a systematic review [18]. This may be because nurses with more years of experience have a deeper understanding of the importance and benefits of EBP. Moreover, the sample in this study included 12.8% head nurses and 5.2% nurse educators. Further analysis revealed that the proportions of head nurses and nurse educators in the high EBP engagement group (15.5% and 6.5%) were higher than those in the moderate engagement group (10.3% and 3.8%) and the low engagement group (7.0% and 2.3%). Madu and Ajibade proposed that nurses in leadership positions (e.g., head nurses) have access to more position‐endowed resources, including training programs and research support, to enhance their own EBP competencies [19]. These nurses can significantly facilitate the translation and implementation of EBP through transformational leadership, mentorship, and resource integration [19–21]. The accumulation of work experience may be positively associated with professional title promotion and position advancement. Nurses with advanced age and more work experience are more likely to hold middle to senior professional titles and concurrently assume leadership or educational roles, which inherently require greater EBP engagement. A study by Zhang et al. [22] also demonstrated that nurses who were older and had middle to senior professional titles exhibited a relatively elevated level of implementation climate. Therefore, there may be a potential interaction between age/tenure and professional roles (positions/titles), which needs to be verified by further research, and may represent a mechanism contributing to the enhanced EBP capabilities observed in this nurse population. In addition, the results of this study revealed that the EBP implementation of clinical nurses is related to gender, which suggests that attention should be given to the possible differences in EBP cognition and practices among nurses of different genders in terms of nursing management and education. In the future, the underlying reasons for this gender difference can be further explored, such as whether it is related to career development paths or personal learning preferences, to formulate more targeted EBP promotion strategies.

Multivariate logistic regression analysis revealed that EBP beliefs and organizational cultural readiness are influencing factors of EBP implementation. EBP beliefs represent an individual’s perception of the value of EBP and their ability to implement it [14], serving as the intrinsic driving force for practicing EBP. This study revealed that nurses with higher EBP belief scores were more inclined to implement EBP. Mehra et al. [24] also revealed a correlation between EBP beliefs and implementation. It is hypothesized that the reason may be that when nurses firmly believe that EBP can improve patient outcomes and enhance nursing quality, they will overcome obstacles in the process and demonstrate stronger initiative in practice. EBP organizational culture and readiness aim to identify organizational characteristics that are strengths and opportunities for promoting EBP within a healthcare system [25]. It encompasses the organizational cultural context, resource provision, and leadership support [14], constituting the critical external environment that supports nurses’ EBP behaviors. In an organization with a positive culture and high readiness, nurses may perceive stronger organizational empowerment, thereby promoting EBP implementation. The results of this study are consistent with the findings of multiple existing studies [26–28]. Furuki et al. [29] reported that the work environment affects nurses’ EBP knowledge and skills, which are directly related to their EBP implementation. The results of a qualitative study revealed that a positive organizational environment is a facilitator of EBP [30], and a study in Thailand also demonstrated a positive correlation between organizational culture and EBP [31]. However, it is worth noting that many scholars believe that without a supportive organizational culture, even with strong individual beliefs, EBP behaviors are highly susceptible to setbacks [25, 32, 33]. Melnyk et al. [14] pointed out that an EBP organizational culture is a key variable between EBP beliefs and implementation. It can thus be inferred that EBP beliefs and EBP organizational culture do not act independently on EBP implementation; instead, there is likely a profound interaction between them. However, this inference requires further research for verification. Therefore, to improve nurses’ EBP implementation, the focus must be placed simultaneously on strengthening their intrinsic beliefs and optimizing the external organizational environment. On the one hand, practitioners should be encouraged to build beliefs; on the other hand, a positive and supportive implementation environment should be fully provided to ensure the sustainable implementation of EBP.

## 7. Conclusion

This study revealed that the implementation of EBP among clinical nurses in China can be categorized into three distinct groups: low participation, moderate participation, and high participation. Most clinical nurses demonstrated active engagement in EBP implementation. Based on the results of the multivariate logistic regression analysis, EBP beliefs, EBP organizational culture, and readiness were identified as factors associated with clinical nurses’ implementation of EBP. Managers and educators should assist nurses in establishing strong EBP beliefs while concurrently providing a favorable EBP implementation environment, as this approach may prove to be an effective strategy for promoting nurses’ engagement in EBP.

## 8. Limitations

While this study revealed the positive current status and group heterogeneity of nurses’ EBP implementation, it still has certain limitations. First, convenience sampling was used in this study, leading to an unbalanced sample distribution across different regions, and the majority were from tertiary hospitals, which may impose certain restrictions on the generalizability of the study findings. Second, the assessment of nurses’ EBP implementation primarily relied on self‐report questionnaire data, which could be susceptible to reporting bias. Future research could incorporate objective observations of practice behaviors or clinical indicators to conduct a more comprehensive evaluation. In response to the aforementioned limitations, future studies should be conducted on a larger scale, with multicenter collaboration and multidimensional measurements. This approach enables a more accurate portrayal of the current status of nurses’ EBP implementation and its influencing factors, providing a scientific basis for formulating more targeted intervention strategies and thereby continuously promoting the improvement of clinical nurses’ EBP.

## Author Contributions

Yilin Chen: data curation, writing–original draft, and writing–review and editing. Jie Wang: methodology and data analysis. Yi Zhang and Xiuzhu Cao: investigation. Linfang Zhao: supervision and writing –review and editing.

## Funding

No funding was received for this study.

## Conflicts of Interest

The authors declare no conflicts of interest.

## Data Availability

The data that support the findings of this study are available from the corresponding author upon reasonable request.

## References

[1] Balas E. A. and Boren S. A. , Managing Clinical Knowledge for Health Care Improvement, Yearbook of Medical Informatics. (2000) 1, no. 01, 65–70, 10.1055/s-0038-1637943. 27699347

[2] Melnyk B. M. and Fineout-Overholt E. , Evidence-Based Practice in Nursing & Healthcare: A Guide to Best Practice, 2015, 3rd edition, Wolters Kluwer Health.

[3] WHO , European Strategic Directions for Strengthening Nursing and Midwifery Towards Health 2020 Goals, 2017, https://www.who.int/europe/publications/i/item/WHO-EURO-2017-5314-45078-64291.

[4] Qi H. , Yue C. , Dongmei Z. , Xindan Z. , Zhuo Z. , and Xin D. , Application Effect of Evidence-Based Nursing Practice in Related Pressure Injury of Artificial Airway Among Adult ICU Patients, Chinese Nursing Research. (2025) 39, no. 2, 297–302, 10.12102/j.issn.1009-6493.2025.02.019.

[5] Jianli C. , Chunyan W. , Wenjuan W. , Xixi Z. , Mingxi L. , and Weiming H. , Evidence-Based Practice of the Puncture Management in Hemodialysis Patients With Difficult New Arteriovenous Fistula, Chinese Journal of Practical Nursing. (2022) 38, no. 13, 973–979, 10.3760/cma.j.cn211501-20210524-01484.

[6] Hu J. , Zhou C. , Feng L. , and Yang Y. , An Evidence-Based Nursing Practice Affects Stress State, Coagulation, Complications and Quality of Life in Patients With Upper Gastrointestinal Bleeding in the Emergency Department: A Retrospective Observational Study, BMC Gastroenterology. (2024) 24, no. 1, 10.1186/s12876-024-03507-1. PMC11600764 39604901

[7] Wu D. X. , Hu J. X. , Wu X. L. et al., Preoperative Evidence-Based Practice for Prevention of Early Postoperative Infections in Patients Receiving a Liver Transplant, Annals of Transplantation. (2024) 29, 10.12659/AOT.943610. PMC11416044 39285624

[8] Jing Z. , Yajie Z. , Yi W. et al., The Behavior of Evidence-Based Nursing and Its Influencing Factors Among Nurses in Tertiary Hospitals in China, Chinese Nursing Management. (2025) 25, no. 6, 927–931, 10.3969/j.issn.1672-1756.2025.06.024.

[9] Edmealem A. , Fentaw N. , Bekele A. , Tegegne B. , Mohammed J. , and Liknaw T. , Nurses’ Implementation of Evidence Based Practice in Nursing Process and Its Associated Factors in South Wollo Zone Public Hospitals, Northeast Ethiopia: A Mixed Method Study, BMC Nursing. (2024) 23, no. 1, 10.1186/s12912-024-02444-4. PMC11515356 39449058

[10] Dabak Z. , Toqan D. , Malak M. Z. , Al-Amer R. , and Ayed A. , Knowledge, Attitudes, Practice, and Perceived Barriers Toward Evidence-Based Practice Among Palestinian Nurses in Intensive Care Units, BMC Nursing. (2024) 23, no. 1, 10.1186/s12912-024-02646-w. PMC11668080 39716237

[11] Adombire S. , Baiden D. , Puts M. , Puchalski R. L. , Ani-Amponsah M. , and Cranley L. , Knowledge, Skills, Attitudes, Beliefs, and Implementation of Evidence-Based Practice Among Nurses in Low- and Middle-Income Countries: A Scoping Review, Worldviews on Evidence-Based Nursing. (2024) 21, no. 5, 542–553, 10.1111/wvn.12734. 38853345

[12] Yini C. , Bihua K. , Qiong X. , Aiai H. , Xiuxin T. , and Yanli Z. , Analysis on the Status Quo of EBP-Related Knowledge, Attitude and Practice Among Nurses in Primary Hospitals and the Influencing Factors, China Medicine and Pharmacy. (2023) 13, no. 2, 123–127, 10.3969/j.issn.2095-0616.2023.02.031.

[13] Yun J. , Miaolin C. , Yanping Y. , Li Y. , Shuangxia H. , and Juan Z. , The Status and Influencing Factors of Evidence-Based Practice Ability Among Emergency Nurses, Chinese Journal of Nursing Education. (2020) 17, no. 5, 469–472, 10.3761/j.issn.1672-9234.2020.05.019.

[14] Melnyk B. M. , Hsieh A. P. , Gallagher-Ford L. et al., Psychometric Properties of the Short Versions of the Ebp Beliefs Scale, the EBP Implementation Scale, and the Ebp Organizational Culture and Readiness Scale, Worldviews on Evidence-Based Nursing. (2021) 18, no. 4, 243–250, 10.1111/wvn.12525. 34288388

[15] Weihua L. , Wenhui B. , Ying L. et al., Chinesization of the Three EBP Scales-Short Version and Its Reliability and Validity Test, Journal of Nurses Training. (2023) 38, no. 19, 1741–1745, 10.16821/j.cnki.hsjx.2023.19.003.

[16] Wudu M. A. , Tarekegn S. M. , Wondifraw E. B. et al., Uptake of Evidence-Based Practice and Its Predictors Among Nurses in Ethiopia: A Systematic Review and Meta-Analysis, Frontiers in Pharmacology. (2024) 15, 10.3389/fphar.2024.1421690. PMC11291372 39092215

[17] Mengna L. , Anlong W. , Hui X. , and Yiting L. , Status Quo and Influencing Factors of Evidence-Based Nursing Competencies Among Oncology Nurses, Chinese Journal of Modern Nursing. (2024) 30, no. 15, 2051–2055, 10.3760/cma.j.cn115682-20230830-00788.

[18] Gudeta T. G. , Terefe A. B. , Mengistu G. T. , and Sori S. A. , A Systematic Review and Meta-Analysis of Evidence-Based Practice and Its Associated Factors Among Health Professionals in Ethiopia, BMC Health Services Research. (2024) 24, no. 1, 10.1186/s12913-024-11957-2. PMC11608491 39616330

[19] Madu C. S. and Ajibade V. M. , The Role of Nurse Leadership in the Implementation of Evidence-Based Practices in Oncology Care: A Focus on Spain, Cureus Journal of Medical Science. (2025) 17, no. 5, 10.7759/cureus.84858. PMC12106930 40433021

[20] Elsheikh R. , Le Quang L. , Nguyen N. et al., The Role of Nursing Leadership in Promoting Evidence-Based Nursing Practice, Journal of Professional Nursing. (2023) 48, 93–98, 10.1016/j.profnurs.2023.06.007. 37775247

[21] Välimäki M. , Hu S. , Lantta T. et al., The Impact of Evidence-Based Nursing Leadership in Healthcare Settings: A Mixed Methods Systematic Review, BMC Nursing. (2024) 23, no. 1, 10.1186/s12912-024-02096-4. PMC11221094 38961494

[22] Zhang X. , Peng M. , He M. et al., Climates and Associated Factors for Evidence-Based Practice Implementation Among Nurses: A Cross-Sectional Study, BMC Nursing. (2024) 23, no. 1, 10.1186/s12912-023-01694-y. PMC10801976 38254125

[23] Melnyk B. M. , Tan A. , Hsieh A. P. , and Gallagher-Ford L. , Evidence-Based Practice Culture and Mentorship Predict EBP Implementation, Nurse Job Satisfaction, and Intent to Stay: Support for the arcc (©) Model, Worldviews on Evidence-Based Nursing. (2021) 18, no. 4, 272–281, 10.1111/wvn.12524. 34309169

[24] Mehra M. , Raj C. B. , Sharma R. , Singh A. , and Tiwari S. K. , Factors Associated With Beliefs and Implementation of Evidence-Based Practice Among Nurses: A Cross-Sectional Study, BMC Nursing. (2025) 24, no. 1, 10.1186/s12912-025-03596-7. PMC12269217 40671064

[25] Melnyk B. M. , Hsieh A. P. , and Mu J. , Psychometric Properties of the Organizational Culture and Readiness Scale for System‐Wide Integration of Evidence‐Based Practice, Worldviews on Evidence-Based Nursing. (2022) 19, no. 5, 380–387, 10.1111/wvn.12603. 36053819

[26] Hu S. , Liu S. , Li X. et al., Organizational Evidence-Based Practice Culture, Implementation Leadership, and Nurses: A Bidirectional Mediation Model, International Nursing Review. (2025) 72, no. 2, 10.1111/inr.13054. PMC11969321 39440962

[27] Xiao M. , Zhou T. , and Pei Y. , Analysis of the Current Situation and Influencing Factors of Clinical Nurse Association Standard Evidence-Based Practice, Journal of Nursing Management. (2025) 2025, no. 1, 10.1155/jonm/3313404. PMC12133361 40463514

[28] Yaru Z. , Hong Y. , Yuhan L. , and Qi C. , Current Situation and Relevant Factors of Evidence-Based Nursing Competence of Oncology Nurse Specialists, Chinese Journal of Modern Nursing. (2024) 30, no. 2, 222–227, 10.3760/cma.j.cn115682-20230418-01514.

[29] Furuki H. , Sonoda N. , and Morimoto A. , Factors Related to the Knowledge and Skills of Evidence-Based Practice Among Nurses Worldwide: A Scoping Review, Worldviews on Evidence-Based Nursing. (2023) 20, no. 1, 16–26, 10.1111/wvn.12623. 36571237

[30] Zhang N. , Zhang Q. , Li C. et al., Barriers and Facilitators of Implementation Sustainability of Evidence-Based Practice for Peristomal Irritant Contact Dermatitis: A Descriptive Qualitative Study, International Journal of Nursing Science. (2025) 12, no. 3, 285–292, 10.1016/j.ijnss.2025.04.002. PMC12168460 40529453

[31] Manitkul N. , Thummathai K. , and Bhatarasakoon P. , Factors Related to Evidence-Based Practices Among Mental Health Nurses in Thailand: A Cross-Sectional Study, Nursing Reports. (2024) 14, no. 4, 3084–3096, 10.3390/nursrep14040224. 39449461 PMC11503284

[32] Melnyk B. M. , Gallagher-Ford L. , Zellefrow C. et al., The First U.S. Study on Nurses’ Evidence-Based Practice Competencies Indicates Major Deficits That Threaten Healthcare Quality, Safety, and Patient Outcomes, Worldviews on Evidence-Based Nursing. (2018) 15, no. 1, 16–25, 10.1111/wvn.12269. 29278664

[33] Saunders H. , Gallagher-Ford L. , Kvist T. , and Vehviläinen-Julkunen K. , Practicing Healthcare Professionals’ Evidence-Based Practice Competencies: An Overview of Systematic Reviews, Worldviews on Evidence-Based Nursing. (2019) 16, no. 3, 176–185, 10.1111/wvn.12363. 31074582

